# Extracorporeal membrane oxygenation (ECMO) in pediatric congenital heart disease: a comprehensive review

**DOI:** 10.1186/s43044-025-00630-6

**Published:** 2025-03-24

**Authors:** Muhammad Shaheer Bin Faheem, Hafiza Qurat Ul Ain, Muhammad Haroon-Ul-Rasheed, Rohma Aftab

**Affiliations:** 1 Karachi Institute of Medical Sciences, KIMS - CMH Malir, Karachi, Pakistan; 2 CMH Multan Institute of Medical Sciences, Multan, Pakistan

## Abstract

**Background:**

Extracorporeal membrane oxygenation (ECMO), which provides life-saving assistance in severe cardiac and pulmonary failure cases, has emerged as an important technique in managing children with congenital heart disease (CHD).

**Main body:**

In this review, we discuss the evolution of ECMO over the years, its clinical uses, and the results in pediatric CHD. ECMO has been utilized as a bridge to recovery, in stabilizing an individual after surgery, and as a bridge to heart transplantation. Cannulation procedures that are adjusted according to the anatomy of an individual have improved outcomes, although bleeding and neurologic concerns remain a matter of concern. In addition, long-term neurodevelopmental disorders and renal failure are also among the alarming outcomes. The use of newer anticoagulant drugs like bivalirudin, which lowers the risk of bleeding, and genomic testing for personalized treatment are examples of recent developments. Furthermore, neuroprotective techniques such as erythropoietin and dexmedetomidine can also enhance the neurocognitive outcomes. Finally, improvements in monitoring systems and pump technology contribute to increased ECMO efficacy and safety.

**Conclusion:**

Despite these developments, ECMO’s expense and restricted accessibility remain major obstacles, especially in areas with low resources. In this review, the advancements in ECMO technology and care are highlighted, and it also emphasizes future research to address the current challenges.

## Introduction

Congenital heart disease is the leading cause of significant congenital defects, posing a significant health challenge. Heart defects account for 28% of all major congenital defects and anomalies [1]. Studies around the world report varying rates of congenital heart disease. It is generally agreed that the best estimate is 8 per 1000 live births [2]. Even though there has been a significant improvement in early survival after the surgical operation following structural repair of congenital heart diseases (CHD), a small percentage of children still suffer refractory heart failure despite receiving anatomically adequate correction. An extension of traditional cardiopulmonary bypass approaches, extracorporeal membrane oxygenation (ECMO) assists cardiac or respiratory function for an extended period. It offers physiologic cardiopulmonary support for patients experiencing acute, reversible cardiac or respiratory failure. For mechanical support of heart or lung function for up to 30 days, the term “extracorporeal life support” (ECLS) was put forward. Although ECLS is employed for cardiac and respiratory systems, and ECMO is technically used for techniques that offer a pulmonary support system, which involves oxygenation and carbon dioxide removal, both terms are still used conversely [3, 4]. In the past several years, extracorporeal membrane oxygenation, or ECMO, has been used extensively to treat cardiac and pulmonary failures in patients with CHD following surgical repair [5]. To provide a thorough understanding of pediatric ECMO’s function in treating congenital heart disease in newborns and children, this review will discuss the historical development, clinical indications, sophisticated cannulation procedures, and results of ECMO in this pediatric population.

### Historical evolution of pediatric ECMO

The very first successful neonatal ECMO survivor, infant Esperanza, was reported by Bartlett in 1976. Due to meconium aspiration-induced respiratory failure, she required 3 days of ECMO assistance [4]. In 1985, Dr. Bartlett oversaw the University of Michigan’s prospective randomized, controlled trial (RCT) that compared respiratory ECMO in neonates to traditional treatment. This study concluded that ECMO had a clear advantage, as ECMO-supported patients survived, whereas traditionally treated patients died [6]. The study was heavily criticized. As a result, in 1989, Dr. Pearl O’Rourke conducted a second, more extensive study at Boston Children’s Hospital. Only six of the ten patients who received conventional support made it out alive, but twenty-eight of the 29 patients who received ECMO survived [7]. Later, Mugford and colleagues conducted a Cochrane review in which they compared ECMO and traditional therapy for infant cardiorespiratory failure in four studies. Greater survival to discharge from the hospital with ECMO support was identified in all four studies when compared with standard therapy. Only 44% of the 244 newborns in the conventionally treated group survived, compared to 77% in the ECMO group [8].

### Evolution of ECMO technology

Extracorporeal membrane oxygenation (ECMO), which has been demonstrated to dramatically lower mortality and morbidity in seriously ill infants and adults, has become a standard treatment option in numerous medical facilities for patients who are not responding to traditional therapy for sudden respiratory and cardiac failure [9]. According to a 2008 study of North American active ECMO healthcare facilities, more than 80% consistently employed older roller pumps for newborn ECMO. Despite the fact that centrifugal pumps and hollow-fiber oxygenators were two newer ECMO devices that entered the circuit, many doctors were hesitant to use them because of the possible hemolysis. Majority of facilities continue to use the tradiational roller pumps for ECMO [10]. The use of the earlier centrifugal pumps has had controversial results. According to a 2004 Extracorporeal Life Support Organization registry review, hemolysis happened in 13.6% of all ECMO treatments, with centrifugal pumps having a greater risk of severe bleeding [11]. The mechanics of newer centrifugal pumps that use magnetic levitation (ML) were introduced to allow them to overcome the drawbacks of earlier models [12]. Using standard and state-of-the-art magnetic levitation (ML) centrifugal pumps, the retrospective study sought to evaluate the safety and effectiveness of veno-arterial (V-A) ECMO for cardiac reasons in neonates, babies, and children. A PediVas Blood Pump (Levitronix LLC, Waltham, MA, USA) was part of the ML circuit. A follow-up after 34 months showed that greater hospital survival and a higher late survival were observed in individuals receiving ML assistance. The use of magnetic levitation centrifugal pumps also resulted in improved end-organ recovery [13]. These results indicate that newer technology can be integrated into future practice for better results.

### Indications for pediatric cardiac ECMO

#### Bridge to recovery

For many years, the main assistance method for pediatric patients with end-stage heart failure has been ECMO [14]. ECMO can serve as a link to recovery when a patient’s heart function is likely to recover, and there is a likelihood of reversible underlying disease. ECMO can be utilized as a bridge to VAD support for patients with cardiac illnesses beyond recovery who are eligible for long-term care. Therefore, in the pediatric congenital heart disease (CHD) population, ECMO is utilized to support cardiac or cardiac and respiratory failure caused by hemodynamic instability before operation or post-operative individuals with low cardiac output syndrome (LCOS) [15].

#### Long-term mechanical support

ECMO has been the clinical standard treatment for children who require mechanical circulatory support as a crucial link to heart transplantation for more than 20 years [16]. When traditional therapy fails, ECMO has emerged as a crucial component of the methods available in hospitals for treating children with heart disease to provide them with temporary support. As the body receives a sufficient blood supply, ECMO support aims to give the failing myocardium an opportunity to heal. For neonatal heart disease, ECMO is typically used as long-term support while decision-making (e.g., organ donor and diagnostic work-up) takes place [17]. Children with myocardial failure who underwent heart transplantation were treated with extracorporeal membrane oxygenation (ECMO) at a facility. The length of ECMO was roughly 6.7 days. The follow-up period was 4.3 years on average. Twenty-one patients survived until hospital discharge (84%), while four patients passed away less than a week after HTx. At 1 and 5 years, post-transplant survival rates were 67% and 52%, respectively [16].

#### Post-operative stabilization

Following pediatric heart surgery, the use of ECMO has been growing. Instead of using ECMO as a last resort in management, the results show a significantly higher survival rate when the integrated ECMO-CPB circuit was used as an early intervention [18]. ECMO has also proven to be extremely vital for patients who experience cardiorespiratory failure. Veno-arterial (VA) ECMO’s primary purpose is to produce cardiac output while decreasing the fatigue on the heart after an operation to give the body time to recuperate and bridge itself to recovery [19].

#### Medical heart disease

**Fig. 1:**
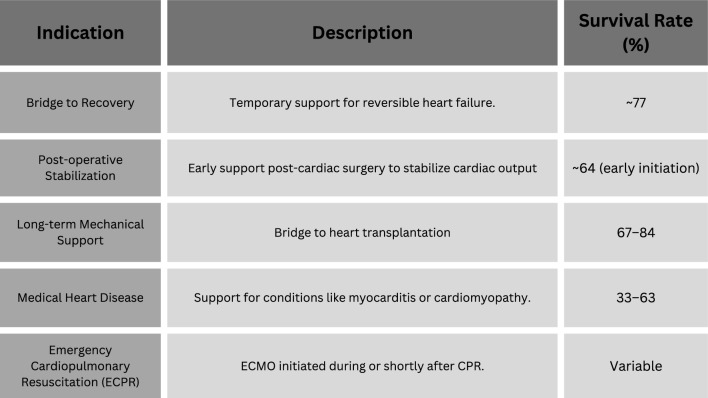
Extracorporeal membrane oxygenation (ECMO) indications and survival rates

While widespread use of extracorporeal life support has been for post-surgical cardiogenic shock, there are other rationales for the use of veno-arterial ECMO in patients with cardiac failure [20]. The therapeutic results of individuals who need ECMO for fulminant myocarditis are encouraging. Teele et al. published a 12-year study of 20 pediatric patients with acute fulminant myocarditis, with 10 ultimately needing VA-ECMO assistance [21]. ECMO has also been used in multiple settings besides the ones mentioned above. In a study, ECMO results in pediatric patients with cardiomyopathy had the best survival to discharge at 63%, followed by those with myocarditis at 59%. Subsequently, 2-CHD and 1-ventricle CHD were at 44% and 33%, respectively [20]. Figure 1 outlines the main indications for ECMO, emphasizing its role in pediatric patients.

#### Patient selection and timing

##### Choosing wisely

When it comes to ECMO cannulation for children/neonates with heart failure and congestive heart failure, there are four primary strategies [15]. ECMO can serve as an intermediary (temporary support) to recovery when a patient’s heart function is likely to recover, and there is a likelihood of reversible underlying disease. ECMO can be utilized as a bridge to VAD (ventricular assist device) assistance for eligible individuals for long-term care. Additionally, ECMO can serve as a stopgap measure before heart transplantation. Lastly, ECMO can aid additional decision-making while obtaining pertinent data or assessing the course of comorbidities in individuals with varying expectations of recovery [15].

##### Pre-operative ECMO support

Sometimes, infants with cardiogenic shock from untreated CHD need to have ECMO started. Tetralogy of Fallot with no pulmonary valve, major Ebstein’s anomaly with a circular shunt, and blocked total anomalous pulmonary venous return are a few rare examples [22–24]. Mascio and colleagues’ research, which examined 96,596 congenital cardiac procedures from 80 institutions, revealed that 0.5% of patients (n = 463) had mechanical circulatory support prior to surgery. In contrast, another 0.1% had both pre- and post-operative assistance [25]. In these circumstances, ECMO is used to increase end-organ blood flow and offer physiologic equilibrium prior to an operation [15].

##### Post-operative ECMO support

Most ECMOs take place in the post-operative phase (2.2%) in comparison with 0.5% before surgery. Mechanical circulatory support rates have been greatest for the Norwood surgery (17.0%), arterial switch operation with a ventricular septal defect and repairing the aortic arch (14.0%), biventricular repairs such as the Ross–Konno technique (9.3%), and truncus arteriosus repair (9.4%) [25]. Other reasons for post-operative ECMO treatment include continuous arrhythmias, such as ectopic junctional tachycardia, which causes hemodynamic dysfunction [15].

##### ECPR

The start of ECMO during standard CPR or under 20 min of spontaneous circulation returning is known as ECPR [26].

##### Timing matters

Most people agree that starting ECMO before significant tissue hypoxia and end-organ damage develop is the optimal course of action. Research has indicated a correlation between poor outcomes in individuals with congestive heart failure and delayed ECMO commencement [27]. According to a study on post-operative cardiac ECMO, patients cannulated in the operating room have a greater survival rate than those cannulated in the cardiac critical care unit. This is because early, adequate assistance helps to prevent protracted hypoperfusion and fatal cardiac collapse. In this instance, ECMO started in the operating room, which resulted in a much greater survival rate (64% vs. 29%) [28].

### Cannulation strategies

#### Peripheral versus central cannulation

**Fig. 2:**
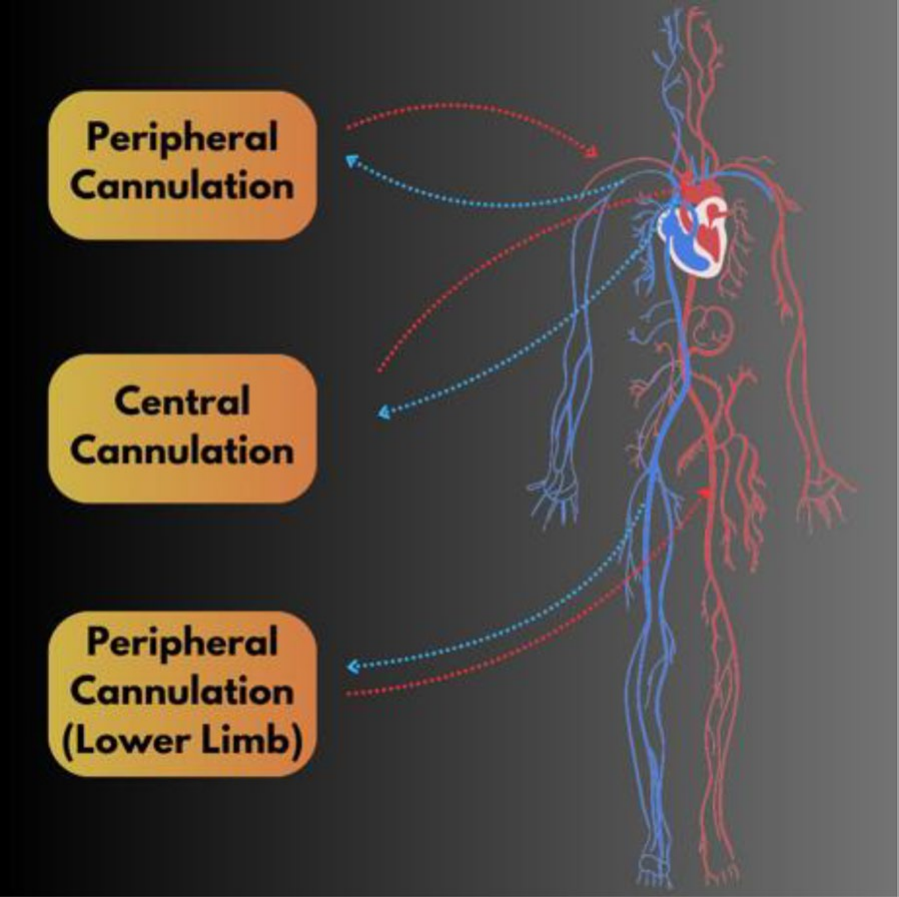
Demonstration of central and peripheral cannulations

The size, previous surgical procedures, and underlying heart anatomy of a patient all influence the cannulation location and ECMO technique for cardiac ECMO. VA-ECMO can be carried out by central cannulation (usually right atrial drainage to the ascending aorta) or peripheral cannulation (drainage through the right internal jugular vein to the right common carotid artery). The cannulation strategy is an important part of ECMO and varies from patient to patient. Figure 2 compares central and peripheral cannulation procedures, showing the venous and arterial cannulation locations for each, and also highlights the peripheral cannulation in groin among the pedetrics patients (less than 16 years, between 20 and 40 kg). In the early post-cardiotomy phase, or if the diameter of the peripheral vessels and the choice of cannula would make it impossible to get sufficient ECMO flow, central cannulation is typically convenient and preferred [29].

Cannulation in the context of ECPR can be peripheral or central, and it should be customized based on the patient’s size, anatomy, and clinical characteristics, such as prior sternotomies and recent heart surgery. Peripheral cannulation was shown to have a greater survival rate in pediatric ECPR cases in one study; however, this finding may have been influenced by the higher percentage of non-operative children in the peripheral cannulation subgroup [30]. Despite being less invasive than central cannulation, peripheral neck cannulation has several significant drawbacks. For the ipsilateral cerebral hemisphere to receive sufficient perfusion from carotid artery cannulation, the Circle of Willis must remain intact. There have been reports of neurologic issues resulting from neck cannulation [31]. Compared to peripheral cannulation, which has higher maximum flow rates, central cannulation typically permits bigger cannulas. A trained team with experience in this cannulation technique is needed for the more invasive central cannulation procedure. Extensive bleeding is one of the most prevalent challenges in the post-operative context [32].

#### Tailoring to individual anatomy

An individual’s previous surgical procedures and underlying heart anatomy all influence the cannulation placement and ECMO technique for cardiac ECMO [29]. In neonates, cannula size and placement present with added difficulties. Smaller veins and cannulation alternatives may make obtaining the intended venous return impossible due to venous drainage problems (if the venous cannula is overly small). Additionally, if the artery cannula is positioned incorrectly or is too tiny, the arterial pressure may increase, resulting in hemolysis and greater post-membrane pressure. Finally, in terms of priming volume, this frequently functions as a newborn’s whole exchange transfusion. The dangers of high potassium, low calcium, and fluid shifts are significant factors to consider while determining the priming amount concerning the neonate’s entire blood volume [33]. In the case of ECPR, it might be quicker to cannulate the right-sided carotid artery and jugular vein in young children who have not had a recent sternotomy than to do a sternotomy and cannulate the central vessels. Some individuals prefer central cannulation due to peripheral vascular stenosis or blockage, intricate cardiac anatomy, larger cannulas requirement, or an earlier sternotomy. Episodes of internal cardiac massage are necessary for ECPR with central cannulation, which might make cannulation more difficult, prevent regular compressions, and call for a different skill set than basic chest compressions. More information is needed to identify the best cannulation method for ECPR. However, in the end, there is currently no unambiguous agreement regarding the best course of action for CHD patients and whether one strategy might be better than the other [26].

### Specific considerations

#### Single-ventricle hearts

Univentricular circulations were formerly regarded as a contraindication to ECMO because early data indicated that individuals with single-ventricle (SV) physiology had a markedly higher risk of death [34]. Nonetheless, due to evidence of the potential advantages of ECMO as a therapeutic rescue, the treatment has become more common during the past few decades. Ravishankar’s study examined the use of ECMO after the first reconstruction phase for hypoplastic left heart and its varying forms and found that over 40% of the population survived to be discharged from the hospital, including all patients who had shunt thrombosis [35]. In Children’s Hospital Boston, a study of infants with shunted single-ventricular physiology who received VA-ECMO from 1996 to 2005 revealed that their overall survival to discharge (48%) was similar to the 41% of all newborn and children’s cardiac VA-ECMO. Although many factors affect the survival of newborns with shunted functionally single-ventricular circulations, data from multiple studies indicate that patients who are cannulated for shunt thrombus and oxygen deprivation fare more effectively in terms of survival than those who are supported for ventricular problems or following a heart attack [36].

#### Superior cavo-pulmonary anastomosis

Mechanical circulatory support (MCS) after later phases of single-ventricle palliation poses particular difficulties that immediately affect the overall results and strategy. The inferior and superior caval circulations are separated due to the Glenn surgery (superior cavo-pulmonary link). Hence, the cannulation technique needs to be carefully considered. It is crucial to understand the individuals’ unique venous network, including any bilateral, right, or left SVCs and vascular integrity. Additionally, proper system venous drainage is necessary for support to be functional. Decompression of the systemic pumping chamber by an IVC cannula may not ensure sufficient venous drainage from that circulation since the SVC is connected to the pulmonary artery [37].

#### Systemic to pulmonary shunting

The form of systemic to pulmonary shunt that is in use is an important factor for analyzing the outcomes of this population. Hesitancy to use ECMO for individuals managed with the modified Blalock-Taussig shunt stemmed from the possibility that the shunt runoff might not provide enough support for systemic perfusion; to successfully maintain a stable circulation, while on mechanical aid, efforts have concentrated on shunt clipping, which limits pulmonary blood flow and raises ECMO output at the risk of excessive pulmonary circulation. Four individuals underwent early attempts to seal the shunt using medicines during ECMO; however, none of these patients survived. However, mortality was much reduced by 20% in the five patients whose shunt was left patent and whose ECMO flow was raised to account for the shunt runoff [29].

#### Neurodevelopmental outcomes

Neonates with congenital or later-onset cardiac disease are susceptible to long-term abnormalities in a variety of neurocognitive domains, including executive functioning, focus, memory, and visuospatial skills, according to several long-term research. Consequently, survivors of heart failure in the first few weeks of life are more likely to experience academic challenges, similar to individuals with acute respiratory failure [38, 39]. It is mostly unknown if individuals receiving ECMO are particularly more vulnerable to these chronic neurodevelopmental issues. Because of circulation problems following heart repair, patients requiring ECMO reflect a negative selection of cases.

Very few publications compare the neurologic outcomes of patients treated with ECMO against those not treated with ECMO following severe cardiac failure. Tindall et al., on this subject, compared the cognitive outcomes of children aged 4–6 who received ECMO after congenital heart defect surgery to those who did not get ECMO and normal controls. Both patient populations’ general cognitive abilities fell within the normal range, although ECMO-treated patients’ abilities were noticeably worse than those of healthy control subjects. In contrast with cardiac controls and healthy children, the ECMO group performed considerably worse on visual recall; spatial organization specialized neuropsychological tasks and left-hand motor function [40]. However, it was noted that long-term focus and attention, memory for language, general ability to speak, right-hand motor abilities, and perception of touch did not differ between groups [40].

## Outcomes and complications

### Outcomes

When cardiopulmonary bypass (CPB) weaning fails and a pediatric patient’s condition deteriorates in the intensive care unit, PC-ECMO is a useful technique for aiding them. Even though ECMO appears to increase survival among individuals, mortality and morbidity are still significant, particularly in infants with univentricular physiology. Furthermore, genetic conditions should not be regarded as a contraindication for ECMO [41]. A meta-analysis that included pediatric and neonatal patients concluded that, out of the 2683 patients who underwent post-cardiotomy ECMO, the overall survival was 43.3%. Survival by PC-ECMO indications was 37.6% for cardiac arrest, 47.3% for low cardiac output syndrome, and 44.6% for CPB weaning failure. The risk ratio was 0.74 for survival in patients with univentricular physiology, whereas the risk ratio for predisposing genetic conditions was 0.93 [41].

### Short-term complications

It was identified that 7.4% of the problems that young patients who had ECPR experienced and reported to ELSO between 2015 and 2020 satisfied the criteria for fatal brain injury. Brain death rates were lower (less than 2%) in pediatric patients receiving ECMO for either cardiovascular or respiratory reasons without undergoing ECPR. In the neonatal demographics, brain death is less frequently documented; it ranges from 0.2% to 0.7% for all ECMO cases. Another frequent issue that affects infants and newborns receiving ECMO is renal impairment. According to the ELSO, renal dysfunction affects at least 40% of pediatric patients and is indicated by acutely raised creatinine levels or the need for renal replacement. In particular, 29.7–32.2% of adolescent patients need kidney replacement treatment while receiving ECMO, while it was 27–36% of newborns [42].

### Long-term complications

**Fig. 3:**
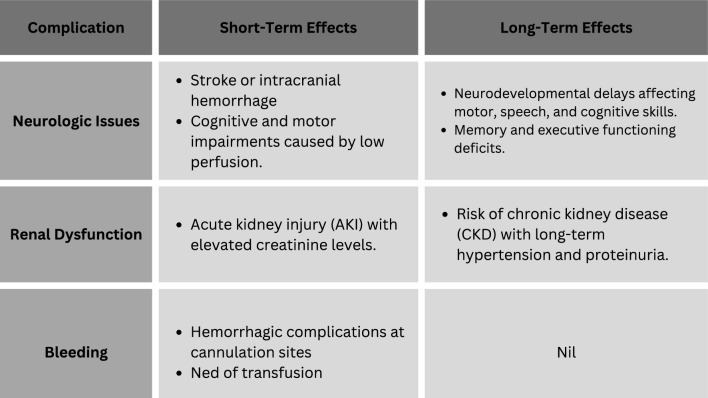
Complications related to ECMO

Patients undergoing cardiac ECMO in research exhibited neurodevelopmental scores at least one standard deviation lower than normal. Individuals performed lower in gross motor function, language, speech, and cognitive functions than the non-ECMO individuals [43]. Interestingly, other studies have made comparisons between VA-ECMO and other mechanical circulatory support techniques and discovered that only 20% of children who survived after ventricular assist device support exhibited the same level of neurologic dysfunction, whereas 60% of children supported with ECMO had moderate to severe neurological problems. Finally, in younger individuals with more complicated congenital cardiac disease, the use of ECMO is linked to poor neurologic consequences [14]. It is unclear what caused this disparity, whether it was because those patients tended to be older and less risky or because they needed less anticoagulation with ventricular assist devices. Even though ECMO has increased survival, there are some risks involved. A brief description of these major complications related to ECMO is given in Fig. 3.

### ECMO in intensive care units during gestation

Cardiac failure that can progress to cardiogenic shock can occur in pregnant women, even if they do not have any risks. A heart attack, arrhythmias, and myocarditis could be the cause of this. The demands of the mother and fetus cannot be satisfied in this situation since the heart would not be able to pump blood normally. ECMO can aid in providing oxygen and circulatory assistance in this situation [44]. This intervention is pivotal for the mother’s and the fetus’s health. In order to decrease complications associated with neonatal prematurity, it may be desirable to continue the pregnancy with the help of ECMO support after the maternal cardiopulmonary disturbances are stabilized [45]. A recent systematic review confirms that it is feasible to prolong pregnancy after the introduction of ECMO. In order to guarantee appropriate oxygenation during ECMO during pregnancy, any degree of hypoxemia is intolerable. For the fetus to receive enough oxygen, the mother’s PaO2 must be maintained at 60 mmHg or an oxygen saturation level higher than 90 percent [46]. Overall, there is disagreement over the best anticoagulation plan for pregnant ECMO patients, with bleeding continuing to be the most common side effect. The most common anticoagulant used systemically during ECMO continues to be unfractionated heparin [47].

Finally, newer advanced techniques such as near-infrared spectroscopy (NIRS) to identify brain damage have been tested in ECMO individuals. Hunt et al. conducted research to identify acute brain damage in V-A ECMO by evaluating the equilibrium between cerebral oxygen supply and consumption using regional oxygenation tissue saturation. Brain damage was indicated by a reduction in regional oxygen saturation by reduction of 25% below the reference [48]. Similarly, urine output is correlated with renal NIRS readings. As urine flow decreases, lower levels are observed, which comes before a reduction in MAP. In neonates, renal NIRS may be a promising non-invasive method for assessing the sufficiency of renal perfusion and urine production [49].

## Future perspectives and research

### Refining ECMO protocols

There are certain neonatal circuit considerations to talk about because neonates make up the bulk of patients with congenital cardiac disease who received ECMO. A recent study comparing the two pump designs in neonates showed that the roller pump group had better results than centrifugal pumps. However, extending this data to our population has significant drawbacks [50]. It is to be noted that the pump used in the research was primarily a roller in the early stages and a centrifugal pump in the later stages. This relates to the hurdles associated with the new method regarding institutional mechanical circulatory support. Furthermore, hemolysis, which was difficult to define in the research due to data constraints, was the cause of the less favorable results.

Considering the necessity of preventing circuit thrombosis, anticoagulation is still complicated. Although there is little prior experience with ECMO in pediatric patients, recent research has demonstrated encouraging outcomes in using bivalirudin (direct thrombin inhibitor). In a study of pediatric individuals receiving ECMO, 16 patients received bivalirudin while 16 received heparin. It was found that the bivalirudin group saw reduced bleeding [51].

Pediatric anticoagulant monitoring has unique difficulties. The scenario is further complicated by the fact that infants with CHD on ECMO are frequently neonates with underdeveloped coagulation networks and may have illnesses that contribute to protein and factor depletion. Activated partial thromboplastin time (aPTT), activated clotting time (ACT), or anti-factor Xa (anti-FXa) levels are used to measure heparin; however, ACT and anti-FXa have a weak correlation, and the link between ACT and dose of heparin is altered by antithrombin III. Additionally, the validity of those tests varies with age [52]. APTT and diluted thrombin time are used to quantify the anticoagulant impact of bivalirudin; however, to keep aPTT within the limit, bivalirudin dosage must be gradually raised. It is noteworthy that bivalirudin will also prolong the INR. Although further research and clinical data are required, ROTEM (rotational thromboelastometry) has been a helpful tool in assessing the impact of bivalirudin in pediatric patients on ECMO [53].

Even though ECMO greatly increases the survival chances of children after cardiac arrest, its high cost and restricted accessibility are significant obstacles. Survival of these individuals can increase if availability and affordability are addressed.

### Neuroprotection strategies

In the newborn intensive care unit (NICU), two possible neuroprotective medications are already being considered for use. Dexmedetomidine, a drug, was seen to have protective benefits on the brain’s hippocampus, specifically against ischemic damage, and is specifically utilized for sedation in the intensive care unit for the children population [54]. It has been proposed that these outcomes stem from dexmedetomidine’s stimulation of α2-adrenergic receptors, which reduces inflammation after cerebral ischemia [55]. This particular method of action is important because it has been discovered that the hippocampus is susceptible to inflammation, hypoxia, and ischemia. Nevertheless, most of these conclusions are derived from research on adults and experimental animals [54, 55]. Erythropoietin is another compound that might be useful in this regard. In the developing brain, several cells create erythropoietin, which has a neuroprotective effect. According to a recent study, a single dose of erythropoietin administered to 6-day-old rats at the beginning of hyperoxia (at 80% oxygen) improved memory deficits and decreased oligodendrocyte breakdown until the teenage and adult stages [56].

Although many drugs have shown potential as neuroprotective medicines in the NICU, most available data comes from adult and animal studies. Further studies in the pediatric population should be encouraged to verify their use and safety in neonates.

### Personalized ECMO approaches and role of genomics

In severely ill newborns, especially those needing ECMO, comprehensive genetic testing using whole-exome (WES) or whole-genome (WGS) sequencing can refine treatment, speed up diagnosis, and perhaps improve the prognosis. A cross-sectional study was carried out in the Children’s Hospital Neonatal Consortium’s Level IV neonatal critical care units in the USA. The extent of the disease, worrying phenotypes, unanticipated postnatal clinical course, and incapacity to wean off of the ECMO were common reasons for WES and WGS. The most frequent reason for genetic testing was an unanticipated degree of severity. Sixty-three percent of facilities supported implementing genetic screening for neonates following ECMO cannulation if quick WES or WGS were easily accessible. Finally, it was concluded that the main obstacle in conducting tests was its expense [57].

For critically unwell neonates on ECMO, genetic testing provides hope by enhancing diagnosis and directing treatment. Increasing the accessibility and affordability of these tests may aid in the better understanding and treatment of the diseases for these infants.

### Global access and equity

A study conducted across the US from the data collected from the NIS (national inpatient sample) concluded that children experiencing cardiac arrest had a greater in-hospital survival rate when the facility had ECMO capabilities. A total of 1276 admissions for cardiac arrest were found. The survival rate was 32% at non-ECMO hospitals and 50% at hospitals with ECMO capabilities. With an odds ratio of 1.49 [95% CI 1.09, 2.02], receiving care at a medical facility with ECMO was linked to a higher in-hospital survival rate [58].

Besides the access to ECMO, the cost of ECMO and related facilities is a huge hurdle for hospitals. ECMO remains an expensive remedy; according to a US study, for a typical hospital stay of roughly 45 days, the average in-hospital expenses for pediatric ECMO patients rose from $214,046 to $324,841 [59].

**Fig. 4:**
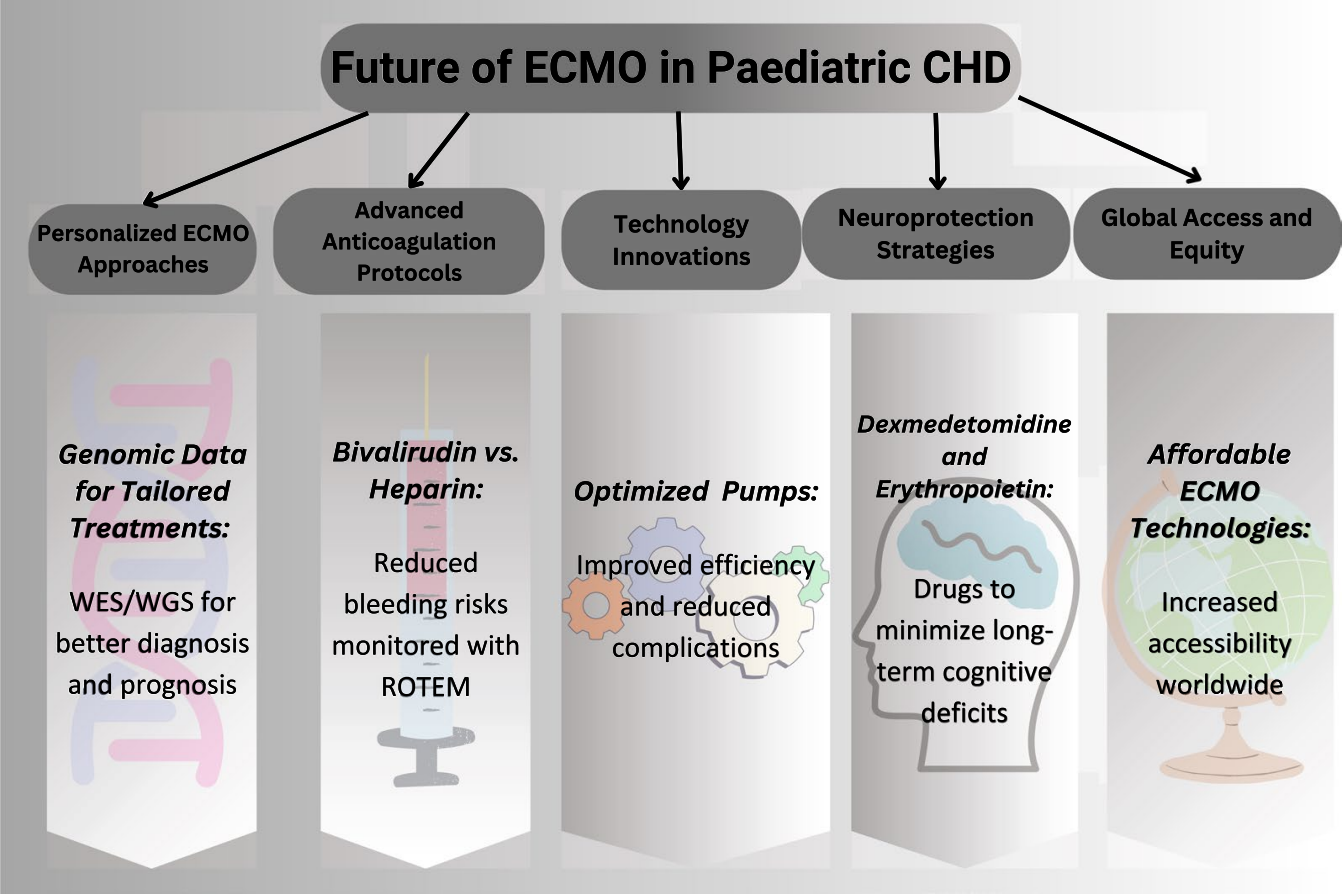
Future directions in pediatric ECMO: demonstrating the junction of technological advancements, personalized medicine, and neuroprotection strategies to enhance outcomes in pediatric patients requiring (ECMO) support

ECMO has proven critical in many situations, providing hope where other options are scarce. However, its high price and restricted availability continue to be a major problem. Improving the affordability and accessibility of this life-saving technology could be critical in the future. Potential future developments in ECMO that could assist pediatric patients are illustrated in Fig. 4.

## Conclusion

In conclusion, ECMO has become an essential tool for critically ill newborns with congenital heart disease, offering crucial support during heart failure, surgeries, and other severe medical conditions. Even though survival rates have increased with the use of ECMO, problems, including long-term development, expensive costs, and timely intervention, still exist. Continued research and improved revised ECMO protocols are critical for the survival of the concerned population at risk.

## Data Availability

No datasets were generated or analyzed during the current study.
